# Single-fraction stereotactic radiotherapy for lung malignancies: preliminary results of a mono-institutional experience

**DOI:** 10.3389/fonc.2026.1933254

**Published:** 2026-08-19

**Authors:** Francesco Cuccia, Gianluca Mortellaro, Marina Campione, Vanessa Figlia, Antonio Spera, Daniela Cespuglio, Salvatore D’Alessandro, Claudio Napoli, Francesco Azzarello, Francesco Verderame, Giuseppe Failla, Livio Blasi, Giuseppe Carruba, Giuseppe Ferrera

**Affiliations:** 1 Radiation Oncology, Azienda di Rilievo Nazionale ad Alta Specializzazione (ARNAS) Civico Hospital, Palermo, Italy; 2 Clinical Research, Azienda di Rilievo Nazionale ad Alta Specializzazione (ARNAS) Civico Hospital, Palermo, Italy; 3 Medical Physics, Azienda di Rilievo Nazionale ad Alta Specializzazione (ARNAS) Civico Hospital, Palermo, Italy; 4 Medical Oncology, Azienda Ospedali Riuniti (AOOR) Villa Sofia-Cervello, Palermo, Italy; 5 Pneumology, Azienda di Rilievo Nazionale ad Alta Specializzazione (ARNAS) Civico Hospital, Palermo, Italy; 6 Medical Oncology, Azienda di Rilievo Nazionale ad Alta Specializzazione (ARNAS) Civico Hospital, Palermo, Italy

**Keywords:** lung cancer, lung metastases, SBRT, single fraction, stereotactic radiotherapy

## Abstract

**Background:**

The use of stereotactic body radiotherapy for primary and secondary lung lesions is well established. Although being supported by international guidelines, the application of single-fraction treatments is less reported in the literature.

**Methods:**

We report a mono-institutional, retrospective analysis of inoperable patients with small lung lesions—including both primary tumors and oligometastases—treated with single-fraction SBRT (SF-SBRT) via a helical tomotherapy linac. Although analyzed retrospectively, all data were prospectively collected. Acute and late toxicities were graded using CTCAE v5.0, while local control (LC), distant progression-free survival (DPFS), and overall survival (OS) were estimated via Kaplan–Meier and Cox regression models.

**Results:**

From August 2023 a total of 50 lung lesions in 49 patients. median age of 77 years (IQR, 72–80) received SF-SBRT. Thirty patients (61.2%) were male. Seven patients (14.3%) had a pacemaker or implantable cardioverter-defibrillator. Of the 50 treated lesions, 30 (60.0%) were primary NSCLC and 20 (40.0%) were pulmonary metastases, being colorectal cancer the most frequent non-pulmonary primary tumor. Most lesions were located in the right lung (62.0%) and in the upper lobe (64.0%). The median maximum tumour diameter was 1.9 cm (IQR, 1.6–2.4), and median prescription dose, for the entire cohort, was 27 Gy (IQR, 26–28), corresponding to a median BED_10_ of 100 Gy (IQR, 93.6–106.4). At a median follow-up of 13.7 months, one local recurrence was observed among the 50 treated lesions (2.0%), distant progression occurred in six of 49 patients (12.2%), while three patients (6.1%) died during follow-up. The estimated 12-month local control, distant progression-free survival and overall survival rates were respectively 100.0%, 89.4% (95% CI, 78.7–100.0%) and 89.5% (95% CI, 78.9–100.0%). No grade 3 or higher adverse events were recorded for both acute and late toxicity patterns.

**Conclusions:**

In elderly patients with a significant burden of comorbidities presenting with isolated lung malignancies, single-fraction SBRT achieved remarkable metabolic responses alongside excellent short-term tolerance and survival. Despite the limitations of a small sample size and few events, these findings support the feasibility and biological effectiveness of single-fraction RT in selected frail subjects, warranting further prospective validation.

## Introduction

The use of stereotactic body radiotherapy (SBRT) for the treatment of lung malignancies is well consolidated. For both patients with early-stage primary lung tumors or patients with pulmonary oligometastases, SBRT is considered as a safe and effective treatment option, supported by international guidelines.

While the majority of literature experiences show excellent outcomes with fractionated regimens of lung SBRT, the use of single-fraction treatments for lung malignancies is less reported (1).

As the ESTRO-ASTRO guidelines suggest the use of single-fraction SBRT (dose range 28 Gy-34 Gy) for peripheral or centro-parenchymal T1-T2 early-stage primary tumors, limited data are available in the literature about the application of this approach (2).

The RTOG 0915 randomized trial compared the outcomes of single-fraction SBRT versus multi-fraction schedules collecting a 1-year local control (LC) rate of 97% in the single-fraction arm opposite to a 92.2% of the multi-fraction arm, with no significant differences in terms of adverse events incidence (3).

In a retrospective series of 229 patients, which represents one of the largest series of patients treated with single-fraction SBRT for lung tumors, Videtic et al. reported a 2-years LC rate of 73.8% with a negligible 3.8% incidence of G3 late adverse events (4).

While pulmonary targets might raise some concern for single-fraction treatments due to the motion management issue and the fears for toxicity, the adoption of SF-SBRT could be useful and advantageous for elderly patients with logistical difficulties or severe cardiopulmonary comorbidities (such as the presence of cardiac devices requiring management during radiotherapy), to whom a curative non-invasive treatment could be offered with a single hospital visit (5).

In the case of metastatic patients, SF-SBRT becomes beneficial when combined with concurrent systemic therapies, as it would reduce the need (at times purely precautionary) to suspend the ongoing systemic treatment or to space it out from radiotherapy.

In 2023, our department launched an SF-SBRT program aimed at optimizing curative treatments for lung lesions, particularly for elderly patients facing logistical challenges in accessing hospital facilities. In this study, we present the preliminary results of our experience.

## Methods

This study was approved by our local Ethical Committee, and all patients have been treated in agreement with the principles of the declaration of Helsinki.

From August 2023 to April 2026 a total of 50 lung lesions in 49 patients were treated with SF-SBRT for lung malignancies in our Institution. Although being a retrospective series, data on safety and efficacy were prospectively collected. Written informed consent was acquired from all patients.

The clinical indications for SBRT required fulfillment of the following parameters: a diagnosis of medically inoperable early-stage non-small cell lung cancer (NSCLC) or isolated pulmonary lesions (up to 3) in oligometastatic individuals, a maximum tumor diameter of 5 cm, a Karnofsky performance status of 70 or higher, and an estimated life expectancy exceeding 6 months. Central or ultracentral lesions were excluded from SF-SBRT, while chest wall proximity was not considered a mandatory exclusion criterion.

The primary objective of this investigation was to assess the feasibility of SF-SBRT in treating both primary and secondary lung tumors.

Secondary outcomes of interest included local control (LC), progression-free survival (PFS), and overall survival (OS).

In the case of early-stage NSCLC, SF-SBRT was proposed for T1-T2 lesions with a peripheral or parenchymal location, while for central or ultra-central targets, a multifraction treatment was proposed, either 50 Gy in 5 sessions or 60 Gy in 8 sessions.

The diagnostic staging protocol involved bronchoscopy, contrast-enhanced total body computed tomography (CT), and 18-fluorodeoxyglucose positron emission tomography (FDG-PET).

In cases presenting with critical comorbidities, histological biopsy was omitted; under these specific circumstances, and in accordance with established literature, positive metabolic imaging was utilized as a surrogate marker for malignancy.

Baseline assessment of lung oligometastases consisted of contrast-enhanced total body CT or PET-CT scans, where needed.

### Radiotherapy protocol

For treatment planning procedures, a 2-mm slice thickness thorax CT scan was acquired in supine position with both arms above the head; to minimize thoracic motion, a chest compression was performed with the ZiFix Thoracic Motion Control System (QFix, Avondale, PA, USA).

The same procedure was performed for the acquisition of a megavoltage computed tomography (MVCT), a slow CT imaging able to provide information about the magnitude of target motion due to respiration (6, 7).

Image fusion of CT with MVCT was performed and clinical target volume (CTV), equal to gross tumor volume (GTV) was delineated in both imaging series (CTV-CT and CTV-MVCT). The internal target volume (ITV) was defined as the sum of the two CTVs. The PTV was generated using an 8 mm cranio-caudal margin and a 5 mm isotropic margin in the remaining directions.

Dose constraints for organs-at-risk and prescription doses were derived from peer reviewed literature and international guidelines (8, 9).

Primary histology (especially in the case of oligometastases) and target location were considered for a risk-adapted strategy of dose prescription.

Treatment planning was performed in order to guarantee a minimum coverage of the 95% of the PTV by the 95% of the prescribed dose, with no more than the 2% of the PTV to receive the 107% of the prescribed dose, still prioritizing healthy structures in the case of close proximity.

All patients received SF-SBRT with Radixact system (Accuray, Sunnyvale, CA, USA). For treatment delivery, the total dose was divided into two mini-fractions, each preceded by a MVCT for IGRT, resulting in a total time slot of 20 minutes per patient.

All patients were prescribed premedication with 25 mg of prednisone, starting the day before the treatment session and continued for 5 days, followed by a subsequent tapering schedule. For patients with a pacemaker or implantable cardioverter-defibrillator (ICD), a cardiological consultation was performed to manage the device during the radiotherapy session.

### Follow-up and statistical analysis

Follow-up imaging consisted of contrast-enhanced total body CT after 3 months from the end of treatment and a PET-CT scan after 6 months from SF-SBRT. Afterwards, for early-stage NSCLC, follow-up was scheduled every 3 months for the first two years and biannually from the third year. For oligometastatic patients, imaging was scheduled mainly every 3 months.

Acute and late adverse events incidence was assessed using the Common Terminology Criteria for Adverse Events v.50 (CTCAE v5.0), assuming as acute any event occurring within 90 days from the end of SF-SBRT, and late any event occurring after 90 days from the end of SF-SBRT.

For statistical analysis, continuous variables are presented as medians with interquartile ranges (IQRs), whereas categorical variables are reported as counts and percentages. Technical feasibility was assessed at the treatment-course level and defined as completion of the planned SF-SBRT course in a single session, with delivery of the full prescribed dose and without unplanned interruption, replanning, conversion to a multi-fraction regimen, or technical failure. Feasibility was expressed as a proportion with exact binomial 95% confidence intervals (CIs).

LC was analyzed at the lesion level. Local failure was defined as documented recurrence within the irradiated target. Time to local failure was calculated from the date of SF-SBRT to the date of in-field recurrence. Lesions without local failure were censored at the date of the last local assessment or death, whichever occurred first.

DPFS and OS were analyzed at the patient level. DPFS was defined as the interval from the first SF-SBRT course to the first documented distant progression. Patients without distant progression were censored at the date of the last disease assessment or death, whichever occurred first. OS was calculated from the first SF-SBRT course to death from any cause or last follow-up. For patients treated for more than one lesion, patient-level time-to-event outcomes were indexed to the first SF-SBRT course. LC, DPFS, and OS were estimated using the Kaplan–Meier method and presented with 95% CIs. Follow-up duration was estimated using the reverse Kaplan–Meier method. Estimates at predefined time points were reported only when at least one patient or lesion remained at risk.

Among lesions with paired numeric pre- and post-treatment SUVmax measurements, changes in metabolic activity were explored using the Wilcoxon signed-rank test. Complete metabolic responses were described separately and were not assigned to a numeric SUV value. Exploratory univariable Firth-penalized Cox regression models were used to assess associations between selected baseline clinical or treatment characteristics and DPFS. Firth penalization was applied to reduce small-sample bias in the presence of sparse events. Hazard ratios (HRs), 95% CIs, and two-sided p-values were reported. No multivariable model was fitted because of the limited number of distant progression events. These analyses were considered hypothesis-generating, and no adjustment for multiple comparisons was applied. Statistical analyses were performed using R software (version 4.4.0; R Foundation for Statistical Computing, Vienna, Austria).

## Results

A total of 50 lung lesions in 49 patients were included; one patient underwent SF-SBRT for two distinct lesions. Baseline patient, lesion, and treatment characteristics are summarized in **Table 1**. The study population was predominantly elderly, with a median age of 77 years (IQR, 72–80), and 38 patients (77.6%) were aged 70 years or older. Thirty patients (61.2%) were male. Comorbidities were highly prevalent, affecting 48 patients (98.0%). Chronic heart disease was the most common comorbidity, reported in 28 patients (57.1%), followed by chronic obstructive pulmonary disease in 16 patients (32.7%) and respiratory impairment or oxygen therapy in 12 (24.5%). Seven patients (14.3%) had a pacemaker or implantable cardioverter-defibrillator. Of the 50 treated lesions, 30 (60.0%) were primary NSCLC and 20 (40.0%) were pulmonary metastases. Adenocarcinoma was the most common histological subtype, accounting for 35 lesions (70.0%), while colorectal cancer was the most frequent non-pulmonary primary tumor. Most lesions were located in the right lung (62.0%) and in the upper lobe (64.0%). Twenty-seven lesions (54.0%) were classified as centro-parenchymal and 23 (46.0%) as peripheral. The median maximum tumour diameter was 1.9 cm (IQR, 1.6–2.4), and 27 lesions (54.0%) measured 2 cm or less. Median pre-treatment SUVmax was 8.2 (IQR, 6.0–10.9), with data unavailable for four lesions. Concurrent systemic therapy was administered during 12 treatment courses (24.0%). The median prescription dose, for the entire cohort, was 27 Gy (IQR, 26–28), corresponding to a median Biologically Equivalent Dose 10 (BED_10_) of 100 Gy (IQR, 93.6–106.4).

**Table 1:** Baseline patient, lesion, and treatment characteristics.

Characteristic	Value
Patient characteristics (n = 49)	
Age, years	77 [72–80]
Age <70 years	11 (22.4)
Age ≥70 years	38 (77.6)
Male sex	30 (61.2)
Female sex	19 (38.8)
Comorbidities	
Any comorbidity	48 (98.0)
Cardiovascular disease	28 (57.1)
Chronic obstructive pulmonary disease	16 (32.7)
Respiratory impairment/oxygen therapy	12 (24.5)
Diabetes mellitus	4 (8.2)
Gastroesophageal reflux disease	7 (14.3)
Pacemaker or implantable cardioverter-defibrillator	7 (14.3)
Lesion and treatment characteristics (n = 50 lesions)	
Primary NSCLC	30 (60.0)
Pulmonary metastasis	20 (40.0)
Histology	
Adenocarcinoma	35 (70.0)
Squamous cell carcinoma	10 (20.0)
Urothelial carcinoma	2 (4.0)
No histological confirmation	3 (6.0)
Primary tumour type	
NSCLC	31 (62.0)
Colorectal cancer	10 (20.0)
Endometrial cancer	4 (8.0)
Bladder cancer	2 (4.0)
Esophageal cancer	3 (6.0)
Treated lung	
Right lung	31 (62.0)
Left lung	19 (38.0)
Treated lobe	
Upper lobe	32 (64.0)
Middle lobe	2 (4.0)
Lower lobe	16 (32.0)
Lesion location	
Centroparenchymal	27 (54.0)
Peripheral	23 (46.0)
Maximum tumour diameter, cm	1.9 [1.6–2.4]
Tumour diameter ≤2 cm	27 (54.0)
Tumour diameter >2–3 cm	18 (36.0)
Tumour diameter >3 cm	5 (10.0)
Pre-treatment SUVmax	8.2 [6.0–10.9]
Missing pre-treatment SUVmax data	4
Concurrent systemic therapy	12 (24.0)
Prescription dose, Gy	27 [26–28]
BED10, Gy	100 [93.6–106.4]
BED10 <100 Gy	25 (50.0)
BED10 ≥100 Gy	25 (50.0)

For primary tumors, prescription doses ranged between 26 and 30 Gy in one session, with lower doses applied in the case of close proximity to the chest wall and the ribs. For secondary lesions, prescription doses ranged between 20 and 28 Gy depending on lesion location and primary tumor histology.

All planned SF-SBRT treatment courses were completed during a single planned treatment session, for a median overall treatment time of 22 minutes (IQR, 18-27). Technical feasibility was 100.0% (50/50 treatment courses; exact 95% CI, 92.9%–100.0%). No unplanned treatment interruptions, replanning procedures, conversions to a multi-fraction regimen, or technical failures were recorded (**Table 2**).

**Table 2:** Technical feasibility of single-fraction stereotactic body radiotherapy.

Feasibility outcome	Treatment courses, n/N (%)
Completion of planned single-fraction treatment	50/50 (100.0)
Full prescribed dose	50/50 (100.0)
Unplanned treatment interruption	0/50 (0.0)
Replanning required	0/50 (0.0)
Conversion to multifraction treatment	0/50 (0.0)
Technical failure	0/50 (0.0)

Acute toxicity was assessed at the patient level according to the highest CTCAE grade recorded after any SF-SBRT course. Among 44 patients evaluable for acute toxicity, 41 (93.2%) had no acute adverse events. Low-grade acute toxicity was observed in three patients (6.8%), including one Grade 1 and two Grade 2 thoracic pain events. No acute Grade ≥3 toxicity was observed.

Among 44 evaluable patients, we recorded no severe late toxicities over the follow-up period. Interestingly, Grade 1 radiation pneumonitis occurred in only 5 patients (11.4% of cases), highlighting the excellent safety profile of the treatment.

Post-treatment metabolic assessment was available for 28 lesions (56.0%). A numeric post-treatment SUVmax measurement was available for 27 lesions (54.0%), while one lesion achieved a complete metabolic response. No post-treatment metabolic assessment was available for 22 lesions (44.0%). Among the 27 lesions with paired numeric pre- and post-treatment SUVmax values, median SUVmax decreased from 9.5 (IQR, 6.8–11.0) before treatment to 2.8 (IQR, 2.0–3.8) after treatment. The median absolute change in SUVmax was −6.6 (IQR, −7.9 to −2.8), corresponding to a significant reduction in metabolic activity (paired Wilcoxon signed-rank test, p<0.001). The lesion achieving complete metabolic response was not included in the paired numeric SUVmax analysis (**Table 3**).

**Table 3:** Acute and late toxicity profile and post-treatment metabolic response assessment.

Characteristic	Value
Acute toxicity (N = 44)	
No acute toxicity (Grade 0)	41 (9.3)
Any acute toxicity	3 (6.8)
Grade 1	1 (2.3)
Grade 2	2 (4.5)
Grade ≥3	0 (0.0)
Late toxicity (N = 44)	
Any late toxicity	0 (0.0)
Metabolic response assessment (N = 50 lesions)	
Post-treatment metabolic assessment available	28 (56.0)
Numeric post-treatment SUVmax available	27 (54.0)
Complete metabolic response	1 (2.0)
Post-treatment metabolic assessment not available	22 (44.0)

**Figure 1:**
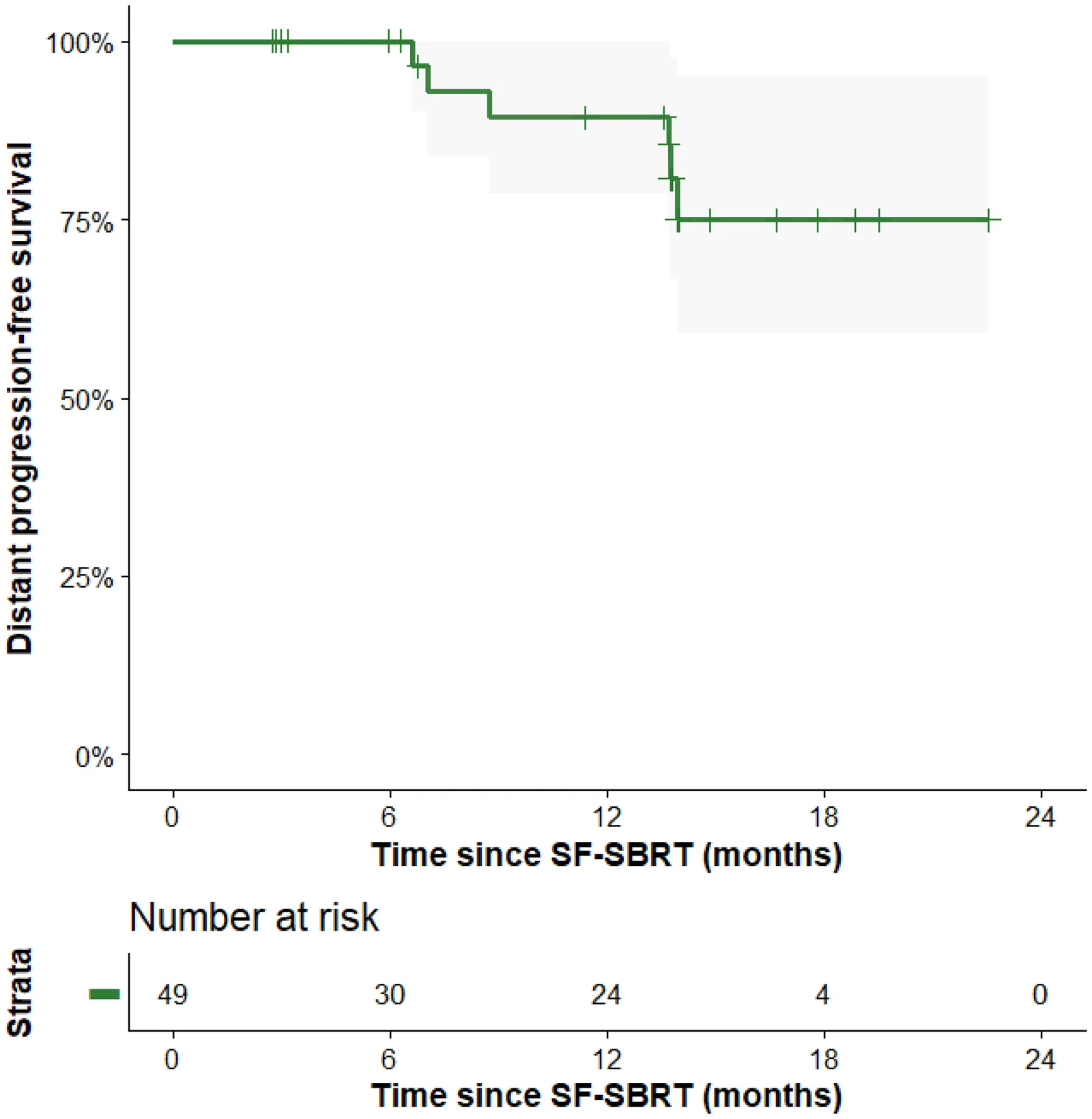
Kaplan–Meier estimate of distant progression-free survival after single-fraction stereotactic body radiotherapy.

**Figure 2:**
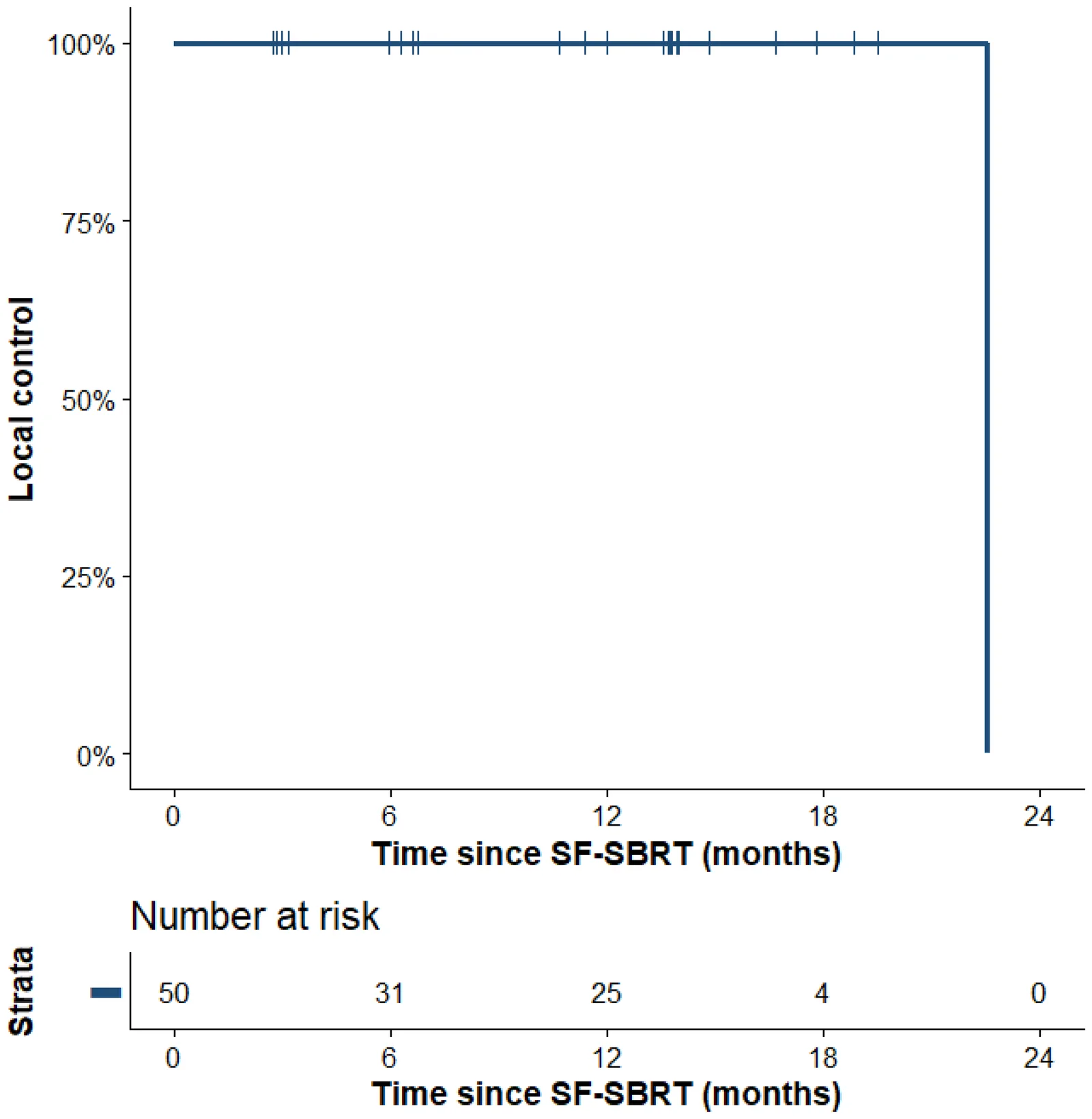
Kaplan–Meier estimate of local control after single-fraction stereotactic body radiotherapy.

**Figure 3:**
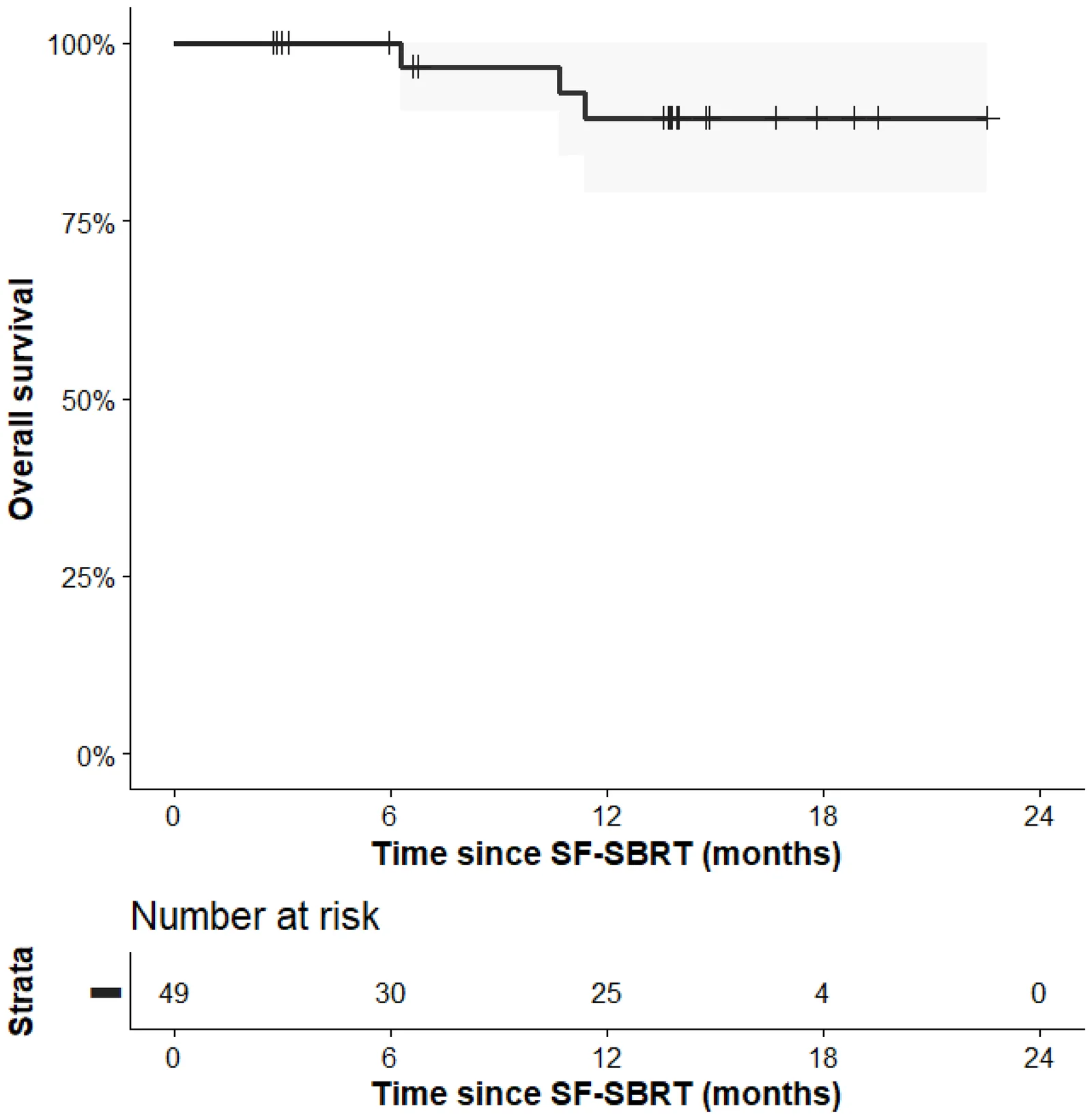
Kaplan–Meier estimate of overall survival after single-fraction stereotactic body radiotherapy.

**Figure 4:**
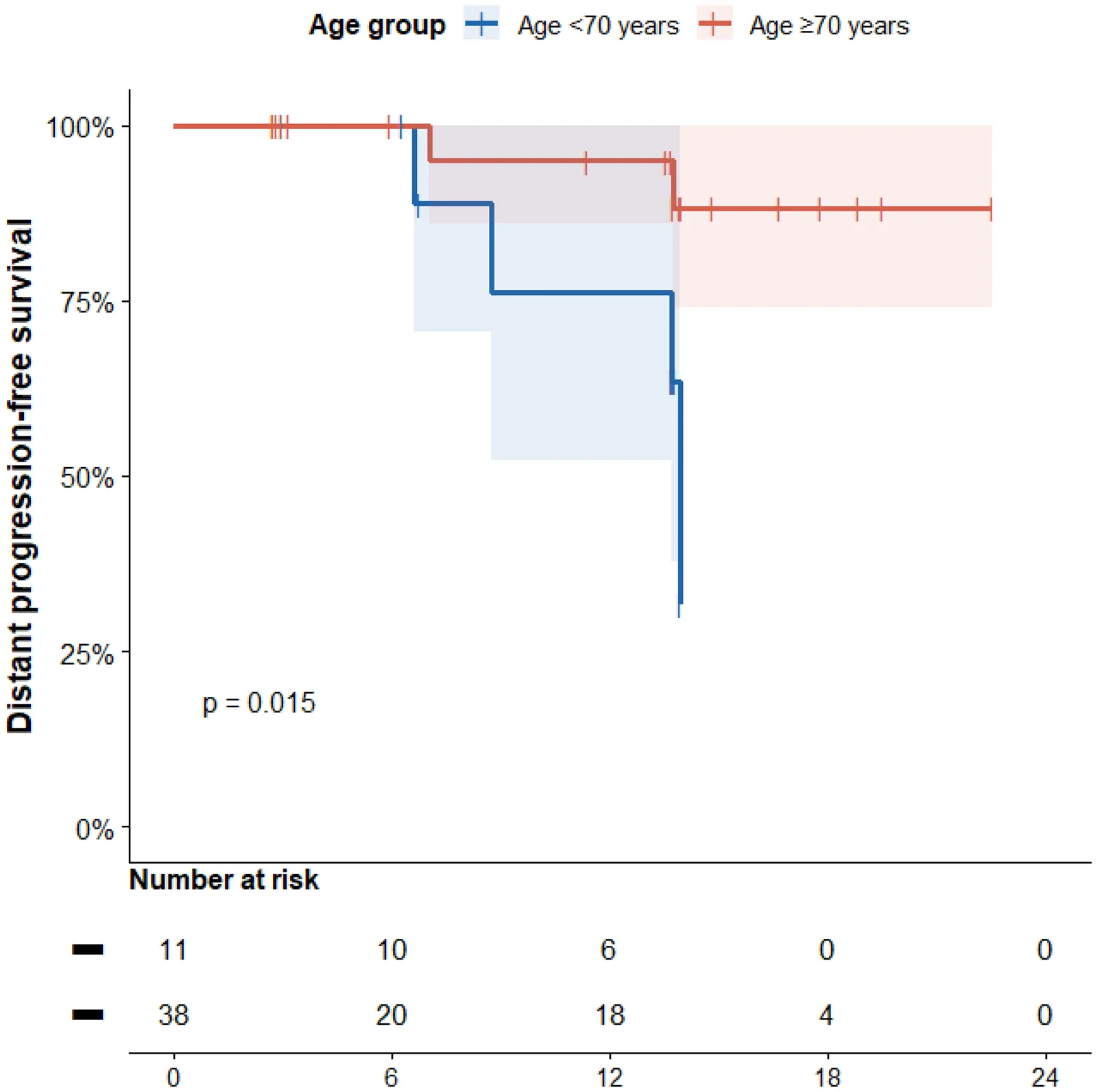
Distant progression-free survival according to age group.

At a median follow-up of 13.7 months, estimated using the reverse Kaplan–Meier method, one local recurrence was observed among the 50 treated lesions (2.0%). At the patient level, distant progression occurred in six of 49 patients (12.2%), while three patients (6.1%) died during follow-up. The estimated 12-month local control rate was 100.0%. At 12 months, distant progression-free survival was 89.4% (95% CI, 78.7–100.0%) and overall survival was 89.5% (95% CI, 78.9–100.0%) (**Figures 1**–**4**).

Exploratory univariable Firth-penalized Cox regression analyses were performed in 49 patients, with six distant progression events. Increasing age was associated with a lower hazard of distant progression (HR per 10-year increase, 0.33; 95% CI, 0.12–0.86; p=0.024). No statistically significant associations with distant progression were observed for sex, cardiovascular disease, chronic obstructive pulmonary disease, respiratory impairment or oxygen therapy, diabetes mellitus, gastroesophageal reflux disease, concurrent systemic therapy, tumour size, or prescription dose. Given the limited number of distant progression events and the wide confidence intervals, these analyses should be interpreted as exploratory and hypothesis-generating (**Table 4**).

**Table 4:** Exploratory univariable Firth-penalized Cox regression analyses for distant progression-free survival.

Covariate	Hazard ratio (95% CI)	p-value
Patient-related factors		
Age, per 10-year increase	0.33 (0.12–0.86)	0.024
Female sex (vs male sex)	1.46 (0.31–6.92)	0.616
Cardiovascular disease (yes vs no)	1.67 (0.33–16.32)	0.56
Chronic obstructive pulmonary disease (yes vs no)	0.31 (<0.01–2.59)	0.338
Respiratory impairment/oxygen therapy (yes vs no)	0.20 (<0.01–1.75)	0.177
Diabetes mellitus (yes vs no)	0.71 (0.01–6.13)	0.807
Gastroesophageal reflux disease (yes vs no)	0.98 (0.01–8.36)	0.991
Treatment- and lesion-related factors		
Concurrent systemic therapy (yes vs no)	2.68 (0.56–12.77)	0.205
Maximum tumor diameter, per 1-cm increase	0.94 (0.35–2.29)	0.901
Prescription dose, per 2-Gy increase	0.67 (0.40–1.32)	0.216

## Discussion

In this study we have summarized the initial outcomes of our mono-institutional program of SF-SBRT for lung malignancies.

In 2023 we started this protocol aiming to improve the logistics for frailer patients with early-stage NSCLC or isolated lung oligometastases. In our perspective, reduced hospital visits favorably impact patients’ quality of life and caregivers’ assistance; in agreement initial experiences, also for other solid tumors, reporting the all-in-one approach as a means to improve both patients’ comfort and treatment cost-effectiveness (10).

Specifically for lung cancer, although being recommended by international guidelines, the use of SF-SBRT is relatively less reported in the literature, likely due to concerns about severe toxicity and the risk of target motion-induced geographical missing (11).

Despite being preliminary, with relatively short follow-up, our initial data support the feasibility and efficacy of SF-SBRT for lung lesions. Our 1-year LC was excellent with no evidence of local failures, with a single case of local relapse, 20 months after SF-SBRT, in a colorectal lung oligometastasis in a 86-years-old patient treated with a dose of 20 Gy.

The known higher radio-resistance of colorectal histology could explain the occurrence of local recurrence in a lesion treated with a BED <100 Gy dose. The patient was one of the first candidates for this treatment and, as part of a learning curve process, we began with escalating prescription doses up to the current 28–30 Gy (12).

Overall, our data is comparable to a similar experience of SF-SBRT by Tekatli et al., where 50 lung primary tumors and oligometastases were treated with MR-Linac. where the authors report a 97% 3-year LC rate, with a single case of G≥3 toxicity (13).

Interestingly, while on one hand respiratory gating with the MR-Linac ensures optimal accuracy during planning and delivery, on the other hand, the authors report an average session duration of 90 minutes, compared to 22 minutes in our series—an important factor to consider regarding the compliance of elderly patients.

Our tolerability patterns are also remarkably favorable with no G3 acute or late toxicity, to date; this might be partially related to the relatively low dose delivered to the target with no patient receiving more than 30 Gy as total dose, and also due to the administration of prednisone in premedication.

Despite its use is not routinely recommended for lung SBRT, as several experiences reported no added value from steroid prophylaxis, literature data mainly refer to multi-fraction treatments, and the RTOG 0236 trial reported as mandatory corticosteroid administration prior to each SBRT session (14, 15).

Considering the prevalence in our cohort of frail elderly patients, we adopted steroid premedication to reduce the risk of acute radiation pneumonitis in such vulnerable patients (16).

**Figure 5:**
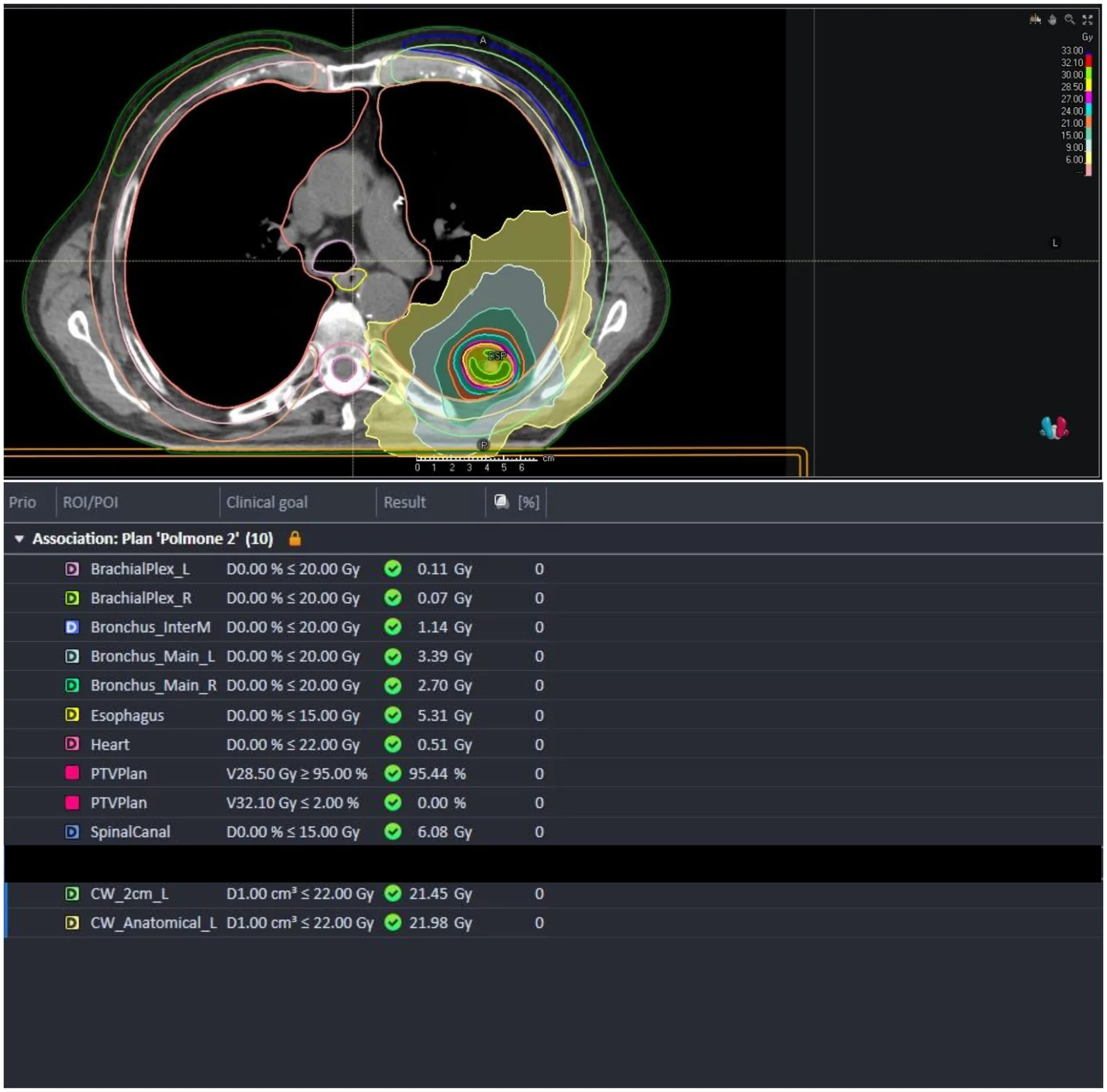
Case example of primary NSCLC of the left lower lobe treated with 30 Gy SF-SBRT with planning goals and dose distribution.

In this regard, another critical issue is the management of cardiac devices, which were present in 14.3% of the patients in our series (**Figure 5**). Delivering radiotherapy without damaging the device represents a crucial challenge for the success of oncological treatment. This must be particularly considered in the case of radiation treatments with very high doses, which can expose the patient to the risk of malfunctions. In these cases, the multidisciplinary involvement of cardiologists, electrophysiologists, and geriatricians is indispensable to allow these patients to receive curative oncological treatment in absolute safety. Despite being an increasingly common occurrence, literature data on patients with implanted cardiac devices are very limited; based on these few cases, a joint position paper by AIRO, AIAC, and AIFM has provided guidelines and recommendations for managing these complex cases (17).

In this scenario, the choice to propose a single-session curative treatment, in addition to reducing patient visits, also minimizes the risk of device malfunctions—an event that did not occur in our series.

This study has several limitations: first of all, it is a retrospective series of both primary and secondary lung lesions, thus leading to a heterogenous cohort. Furthermore, the limited follow-up requires more mature data to deeply understand the real impact of SF-SBRT for lung lesions.

Finally, despite the predominance of elderly patients, a comprehensive geriatric assessment is lacking; nonetheless all patients have been assessed in a multidisciplinary meeting in presence of an Internal Medicine specialist.

However, this study reports promising data for SF-SBRT, demonstrating an encouraging safety and tolerability profile with a negligible incidence of severe side effects. Thus, it represents a valid alternative even for fragile patients, such as the elderly, who are often burdened by significant comorbidities. SF-SBRT serves as an advantageous therapeutic option by reducing hospital visits for these patient categories, benefiting not only the patient but also the caregiver, particularly in cases of significant geographical distance from the hospital facility. Furthermore, although preliminary, the local control data are highly promising, showing results comparable to multi-fraction treatments for both primary and secondary lesions.

## Conclusions

In our experience, SF-SBRT represents a promising and viable option for the treatment of both primary and metastatic lung lesions. Initial results support this approach in terms of safety and efficacy. The use of SF-SBRT could be highly beneficial for more fragile patients facing significant logistical and caregiving challenges.

## Data Availability

The raw data supporting the conclusions of this article will be made available by the authors, without undue reservation.
